# Cytokine-Induced Memory-Like NK Cells with High Reactivity against Acute Leukemia Blasts and Solid Tumor Cells Suitable for Adoptive Immunotherapy Approaches

**DOI:** 10.3390/cancers13071577

**Published:** 2021-03-30

**Authors:** Matteo Tanzi, Michela Consonni, Michela Falco, Federica Ferulli, Enrica Montini, Annamaria Pasi, Rosalia Cacciatore, Silvia Brugnatelli, Paolo Pedrazzoli, Marco Zecca, Stella Boghen, Paolo Dellabona, Giulia Casorati, Daniela Montagna

**Affiliations:** 1 Cell Factory, Fondazione IRCCS Policlinico San Matteo, 27100 Pavia, Italy; m.tanzi@smatteo.pv.it (M.T.); f.ferulli@smatteo.pv.it (F.F.); e.montini@smatteo.pv.it (E.M.); 2 Laboratory of Immunology Transplantation, Fondazione IRCCS Policlinico San Matteo, 27100 Pavia, Italy; 3 Experimental Immunology Unit, Division of Immunology, Transplantation and Infectious Diseases, San Raffaele Scientific Institute, 20132 Milan, Italy; consonni.michela@hsr.it (M.C.); dellabona.paolo@hsr.it (P.D.); casorati.giulia@hsr.it (G.C.); 4 Laboratory of Clinical and Experimental Immunology, Integrated Department of Services and Laboratories, IRCCS Istituto Giannina Gaslini, 16147 Genoa, Italy; michelaemma.falco@gmail.com; 5 Pediatric Hematology Oncology, Fondazione IRCCS Policlinico San Matteo, 27100 Pavia, Italy; m.zecca@smatteo.pv.it (M.Z.); s.boghen@smatteo.pv.it (S.B.); 6 Immunohematology and Transfusion Service and Cell Therapy Unit, Fondazione IRCCS Policlinico San Matteo, 27100 Pavia, Italy; a.pasi@smatteo.pv.it (A.P.); r.cacciatore@smatteo.pv.it (R.C.); 7 Medical Oncology, Fondazione IRCCS Policlinico San Matteo, 27100 Pavia, Italy; s.brugnatelli@smatteo.pv.it (S.B.); p.pedrazzoli@smatteo.pv.it (P.P.); 8 Department of Internal Medicine and Medical Therapy, University of Pavia, 27100 Pavia, Italy; 9 Department of Sciences Clinic-Surgical, Diagnostic and Pediatric, University of Pavia, 27100 Pavia, Italy

**Keywords:** NK-based immunotherapy, haploidentical hematopoietic stem cell transplantation, pediatric leukemia, solid tumors, NK-alloreactivity

## Abstract

**Simple Summary:**

Several strategies have been under investigation on how to enhance anti-tumor responses in patients with poor prognosis. Considering the heterogeneity of antigens expressed by different hematological and solid neoplasia, it is difficult to develop specific immunotherapy approaches. The capacity of Natural Killer (NK) cells to kill tumor cells without specific Ag recognition provides an advantage over T cells and makes them appealing candidate effectors for immunotherapy, even if the effectiveness of their therapeutic use is limited by short-term persistence in vivo. After providing proof of concept regarding the feasibility of inducing NK cells endowed with anti-tumor activity and ability to persist for a protracted period in vivo, considering the relatively short period of in vitro activation, this approach could easily be translated into clinical practice to control tumor growth in high-risk patients.

**Abstract:**

The limited efficacy of Natural Killer (NK) cell-based immunotherapy results in part from the suboptimal expansion and persistence of the infused cells. Recent reports suggest that the generation of NK cells with memory-like properties upon in vitro activation with defined cytokines might be an effective way of ensuring long-lasting NK cell function in vivo. Here, we demonstrate that activation with IL-12, IL-15 and IL-18 followed by a one-week culture with optimal doses of Interleukin (IL-2) and IL-15 generates substantial numbers of memory-like NK cells able to persist for at least three weeks when injected into NOD scid gamma (NSG) mice. This approach induces haploidentical donor-derived memory-like NK cells that are highly lytic against patients’ myeloid or lymphoid leukemia blasts, independent of the presence of alloreactive cell populations in the donor and with negligible reactivity against patients’ non-malignant cells. Memory-like NK cells able to lyse autologous tumor cells can also be generated from patients with solid malignancies. The anti-tumor activity of allogenic and autologous memory-like NK cells is significantly greater than that displayed by NK cells stimulated overnight with IL-2, supporting their potential therapeutic value both in patients affected by high-risk acute leukemia after haploidentical hematopoietic stem cell transplantation and in patients with advanced solid malignancies.

## 1. Introduction

Natural Killer (NK) cells play an important role in anticancer responses based on their capacity to recognize molecular structures that are highly expressed on neoplastic cells [1]. This recognition is regulated by signals triggered by activating and inhibitory receptors expressed on the NK cell surface [2,3]. Over the last few decades, it has been clearly documented that alloreactive NK cells play a crucial role in controlling leukemia relapse in both adult acute myeloid leukemia (AML) [4,5] and pediatric acute lymphoblastic leukemia ALL patients [6], thereby contributing to the efficacy of hematopoietic stem cell transplantation (HSCT) for these malignancies. In addition, alloreactive donor-derived NK cells are able to kill recipient antigen-presenting cells and recipient T lymphocytes, thereby reducing the risk of graft-versus-host disease (GvHD) and graft rejection [7,8,9]. 

Autologous and allogeneic NK cell-based immunotherapies have been evaluated in clinical trials both in hematological and solid malignancies [10,11,12,13,14,15,16]. NK cell-based immunotherapies have generally showed good safety profiles, but their effectiveness in inducing leukemia remission or in controlling solid tumor growth has been limited. The lack of primary NK cell efficacy has been attributed, at least in part, to the lack of antigen specificity and suboptimal expansion/persistence of the transferred cells. Indeed, maintaining proliferative potential and effector functions of the transferred NK cells in vivo remain critical challenges for NK cell therapy [13]. 

In recent years, several groups have reported that mouse and human NK cells can acquire in vivo a so called innate memory-like effector phenotype in response to certain viruses, in particular following CMV infection [17]. Memory-like mouse and human NK cells may also be generated in vitro through combined Interleukin (IL)-12, IL-15 or IL-18 activation [18,19,20,21]. These studies documented that murine memory-like NK cells can still be detected in vivo three months after their transfer and they exhibited potent anti-tumor activity against solid tumors. In addition, it has been demonstrated in a murine model of allogenic HSCT that infusions of IL-12 and IL-18-activated donor NK cells displayed graft-versus-leukemia (GvL) functions and could mitigate GvHD [22]. Recently, a human phase I study demonstrated that infusion of haploidentical memory-like NK cells following chemotherapy to provide immunosuppression and promote engraftment of allogeneic cells was associated with limited toxicity and induced clear signs of clinical response, including complete remission in adult patients affected by AML [23]. Therefore, cytokine-induced memory-like NK cell differentiation ex vivo might be an effective strategy to ensure long-lasting NK effector functions in vivo in refractory patients. However, some questions remain concerning the optimization of the application of this approach in clinical practice in both the autologous and allogeneic setting. In particular, the safety of such therapy for haploidentical HSCT (haplo-HSCT) is uncertain since residual T cells in the final product could induce GvHD, especially if cells are transferred shortly after transplantation. Another critical aspect is whether enough cytokine-induced memory-like NK cells could be obtained at the end of the in vitro culture from a single leukapheresis. Indeed, it is important to emphasize that, particularly in the autologous setting where there is no risk of inducing GvHD, the number of transferred cells could be crucial to achieving a clinical response.

In the present study, we have identified the best culture conditions for ex vivo induction and expansion of cytokine-induced memory-like NK cells that meet two relevant criteria: (1) adequate cellular recovery at the end of the culture and (2) the cells possess effective levels of anti-tumor cytotoxicity. Our study supports the feasibility of inducing ex vivo large amounts of tumor-reactive donor-derived memory-like NK cells for prevention/treatment of leukemia relapse in high-risk children receiving haplo-HSCT or, alternatively, autologous memory-like NK cells for the treatment of patients affected by solid tumors refractory to conventional treatments.

## 2. Materials and Methods

### 2.1. Patients and Healthy Donors

Peripheral Blood Mononuclear Cells (PBMCs) were obtained from five adult patients with solid neoplasia during their follow-up and from 11 hematopoietic stem cell donors during routine analysis for evaluation of donor characteristics in the setting of haploidentical transplantation. Pediatric patients were affected by AML (5 pts) or ALL (6 pts) and were given haplo-HSCT from a partially matched family donor (Table 1). Leukemia blasts (LBs) were derived from bone marrow (BM) at diagnosis (LBs > 90%) during routine analysis and cryopreserved for later use. Adult patients were affected by sarcoma (2 pts) or renal cell carcinoma (3 patients) and underwent surgical intervention to remove the primary tumor and/or metastases (Table 2). All procedures were performed according to guidelines for the treatment of neoplasia, and no patient underwent unnecessary invasive procedures. The study was approved by the Institutional Review Board of Fondazione IRCCS Policlinico San Matteo, Protocol No. 20100041814, April 2011. Tumor cells (TC) were derived from tumor specimens (see the methodology for TC expansion below). NK cells were also isolated from PBMCs derived from buffy coats of healthy donors. Buffy coat units were released by the Blood Bank of SIMT, IRCCS Policlinico San Matteo, Pavia, according to Article 8 of Decree 2, November 2015, of the Italian Department of Health with the blood donor’s specific informed consent as per the Blood Bank Conference of Regione Lombardia.

### 2.2. Analysis of NK Alloreactivity

DNA of donors and patients was extracted using a QIAamp DNA Blood Mini kit (Quiagen, Hilden, Germany). Human Leukocyte Antigen (HLA) typing was performed using the polymerase chain reaction sequence-specific primers (PCR-SSP) technique with Olerup SSP Genotyping commercial kits (Geno Vision, Saltsjoebaden, Sweden) and the Luminex^®^ platform for sequence-specific oligonucleotide-primed polymerase chain reaction (PCR-SSO) [24,25,26]. This technology was applied with LABType^®^ commercial kits (One Lambda Inc.; Canoga Park, CA, USA) and KIR gene profile analysis was performed using an Olerup SSP KIR Genotyping kit (Geno Vision, Saltsjoebaden, Sweden) following the manufacturer’s instructions as previously described [26]. Presence of NK alloreactivity was evaluated using the KIR/KIR ligand mismatch in the graft-versus-host direction model [27].

### 2.3. Expansion of Tumor Cells

Tumor specimens were processed as previously described [28] using a GentleMACS dissociator (Miltenyi Biotec, Bergisch Gladbach, Germany) after treatment with a tumor dissociation Kit (Miltenyi Biotec, Bergisch Gladbach, Germany) according to the manufacturer’s instructions. Tumor cells were filtered to remove clusters, checked for viability with trypan blue die exclusion and resuspended at a concentration of 0.5–1 × 10^6^ cells/mL of the CellGro SCGM (Cell Genix, Freiburg, Germany) supplemented with 20% FBS, 2 mM L-glutamine (complete medium) (Thermo Fisher Scientific, Waltham, MA, USA) and cultured in 25 cm^2^ tissue flasks (Corning, Stone Staffordshire, UK) at 37 °C and 5% CO_2_. Viable tumor cells attached to the flask within 12–24 h. The cultures at 75% to 100% confluence were selected for subculture by trypsinization with 0.25% trypsin and 0.02% EDTA (Life Technologies Inc.) in a calcium/magnesium-free balanced solution. The culture medium was changed twice a week and cellular homogeneity was evaluated microscopically every 24–48 h. The cells were cryopreserved in 90% FBS and 10% DMSO and stored in liquid nitrogen for further experiments.

### 2.4. Experimental Design

**Figure 1:**
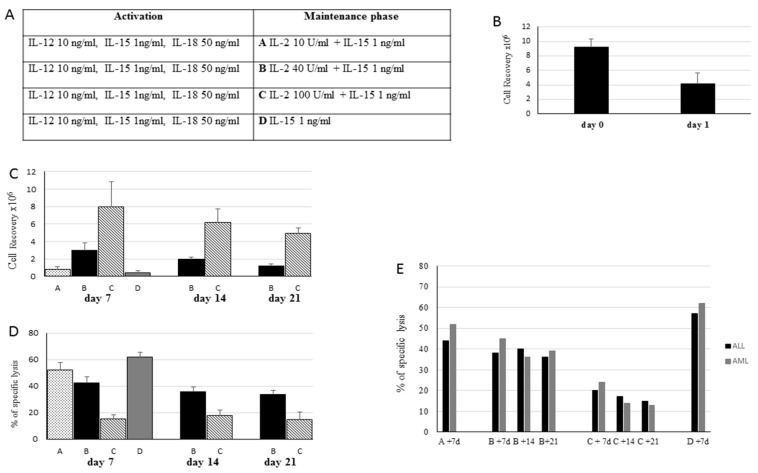
Activation and expansion of memory-like NK cells. (**A**) Scheme of experimental approaches evaluated in the maintenance/expansion phases. (**B**) Recovery of NK cells purified from buffy coats of healthy donors (5) or from PBMCs of haplo-HSCT donors (2) after 16-h activation with IL-12, IL-15 and IL-18. (**C**) Cell recovery after the maintenance phase using different protocols. To better visualize and compare the results, the histograms refer to an initial number of cells of 10^6^. (**D**) Cytotoxic activity of memory-like NK cells isolated from buffy coats of healthy donors recovered at day 7, 14 and 21 and tested in a 4-h cytotoxicity assay against the THP-1 cell line. The means and SDs of percentages of specific lysis at an effector-to-target (E:T) ratio of 30:1 are reported. (**E**) Cytotoxic activity of memory-like NK cells isolated from PBMCs of two haplo-HSCT donors recovered at days 7, 14 and 21 and tested in a 4-h cytotoxicity assay against the respective patients’ LBs. Percentages of specific lysis at an E:T ratio of 30:1 against ALL (black columns) and AML (grey columns) blasts are reported.

**Figure 2:**
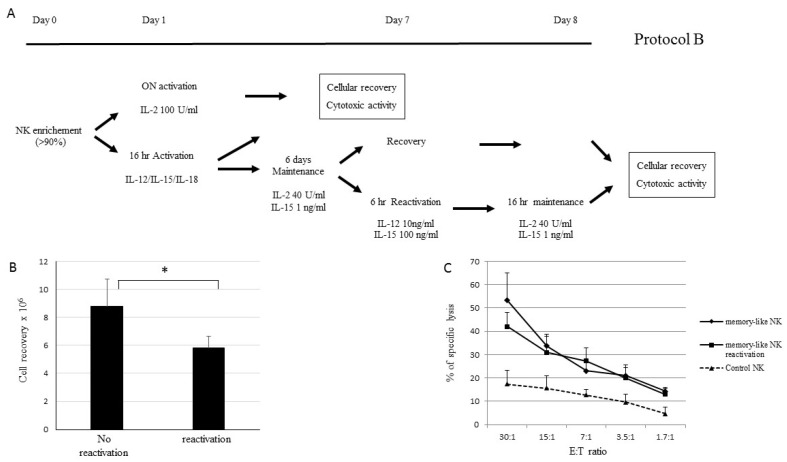
Memory-like NK cells exhibit higher anti-tumor cytotoxicity compared to NK activated ON with IL-2. (**A**) Schematic representation of the approach for ex vivo induction and expansion of memory-like NK cells. The protocol included 16-h activation with IL-12, IL-15 and IL-18 followed by 6-day culture in the presence of 40 U/ml IL-2 and 1 ng/mL IL-15. After 6 days in the maintenance phase, the cells were recovered or alternatively reactivated for 6-h with 10 ng/mL IL-2 and 100 ng/mL IL-15. Alternatively, purified NK cells were stimulated overnight (ON) with 100 U/mL of IL-2 (control NK). After each step, the cells were evaluated for cellular recovery and cytotoxic activity. (**B**) The means and SDs of cell recovery of NK cells recovered at day 7 (no reactivation) or at day 8 after 6-h reactivation and 16-h maintenance (reactivation), * *p* < 0.05. (**C**) The means and SDs of cytotoxic activity of memory-like NK cells obtained at day 7 (♦), at day 8 (■) and of control NK cells (activated ON with rhIL-2) (▲). Results derived from five independent experiments performed with NK cells derived from healthy donors and tested against THP-1 cell lines at various E:T ratios ranging from 30:1 to 1.7:1 are reported.

NK cells purified from the buffy coats of five healthy donors and from leukaphereses of two haplo-HSCT donors using an NK isolation Kit (Miltenyi Biotec, Bergisch Gladbach, Germany) were plated at 3–5 × 10^6^/mL and activated for 16 h in CellGro (CellGenix) supplemented with rhIL-12 (10 ng/mL), rhIL-15 (1 ng/mL) and rhIL-18 (50 ng/mL). After activation, the NK cells were recovered, washed and cultured in CellGro supplemented with IL-2 and IL-15 at various concentrations (Figure 1). On day 7, the NK cells were harvested or, alternatively, maintained in culture in the medium supplemented with the same cytokine concentrations for 14 and 21 days. After each step, the NK cells were analyzed for cell recovery and capacity to display leukemia cytotoxic activity against the THP-1 cell line (human monocyte cell line) and patient-derived LBs (Figure 1). For the maintenance phase, the following different culture conditions were evaluated: (A) 10 U/mL IL-2 + 1 ng/mL IL-15, (B) 40 U/mL IL-2 + 1 ng/mL IL-15, (C) 100 U/mL IL-2 + 1 ng/mL IL-15, (D) 1 ng/mL IL-15 (Figure 1A). Having established the experimental conditions for the optimal induction of sizeable NK cell numbers displaying good levels of anti-tumor cytotoxic activity, all the future experiments adopted this approach. To evaluate the role of NK cell reactivation after the maintenance phase using experimental approach B, the NK cells derived from buffy coats of three different healthy donors were cultured with protocol B, harvested at day 6, reactivated (or not) for 6 h in the presence of 10 ng/mL IL-12 and 100 ng/mL IL-15 and then cultured for 16 h in the medium supplemented with 40 U/mL IL-2 and 1 ng/mL IL-15 (Figure 2). The NK cells incubated overnight with 100 U/mL of IL-2 were defined as control NK cells.

### 2.5. Flow Cytometry Analysis

The Monoclonal antibodies (mABs) used for phenotypic analysis included Fluorescein isothiocyanate (FITC)-, Phycoerythrin (PE)-, Phycoerythrin Cyanine 5.5 and allophycocyanin (APC)-labeled anti-CD45, anti-CD3, anti-CD56, NKp30, NKp44 (Beckman Coulter, Brea, CA, USA), anti-CD16, NKp46, NKG2A (R&D System), anti-CD94, anti-CD69, anti-CD18, anti-CD49, anti-CD62L, anti-CD38 and CXCR4 (BD Pharmingen, San Jose, CA, USA). Phenotype evaluation was performed by direct immunofluorescence according to previously reported methods [29]. NK cells recovered from immunodeficient NOD/SCID gamma (NSG) mice were stained with anti-mouse CD45-APC-Cy7, anti-human CD45-PE-Cy7, CD3-PerCP-Cy5.5, CD56-FITC (Biolegend, San Diego, CA, USA) and CD94-APC (BD).

### 2.6. ^51^Cr Release Cytotoxicity Assay

Target cells included patient LBs, patient TCs, patient Phytohemagglutinin (PHA) blasts, patient bone marrow remission cells (BMRCs) obtained from leukemia patients at remission before haplo-HSCT and the THP-1 leukemia cell line. Cytotoxic activity of NK cells was evaluated in a four-hour cytotoxicity assay against ^51^Cr-labeled target cells at various effector-to-target (E:T) ratios as previously described [30].

### 2.7. Evaluation of IFNγ Secretion

Memory-like NK cells, control NK cells and resting NK cells were analyzed using a Miltenyi Biotec IFNγ secretion assay as per the manufacturer’s instructions. Briefly, the cells were incubated with an IFNγ specific catch reagent at 37 °C to allow cytokine secretion. The secreted IFNγ binds to the catch reagent on the positive secreting cells. Then, the cells were labeled with a second IFNγ-specific detection antibody conjugated to PE (R-phycoerythrin) for detection by flow cytometry.

### 2.8. Evaluation of NK Persistence in Immunodeficient NSG Mice

To obtain an adequate NK cell number necessary to analyze NK persistence in mice, three buffy coats from healthy donors were used for each experiment. NK cells were cultured as follows: for activation: 10 ng/mL IL-12 + 1 ng/mL IL-15 + 50 ng/mL IL-18; maintenance: 6–7 days with 40 U/mL IL-2 + 1 ng/mL IL-15. The results obtained using activated NK cells were compared with those obtained after overnight (ON) activation of purified NK cells with 100 U/mL of IL2 or with 1 ng/mL of IL-15 (control NK). NK persistence was evaluated in 8-week-old NSG mice purchased from Charles River and housed in a specific pathogen-free (SPF) animal facility at the IRCCS San Raffaele Scientific Institute, Milan. All animal experiments were approved by the Institutional Animal Care and Use Committee (IACUC). Briefly, 7 × 10^6^ memory-like NK cells or control purified NK cells were injected i.v. into NSG mice (*n* = 4/group) and their presence in the peripheral blood was evaluated by flow cytometry. The mice were bled from the ocular vein at the indicated timepoints and, after red blood lysis, the cells were stained with the indicated mAbs.

### 2.9. GMP Validation

To confirm the possibility of translating this approach to the clinic, scale-up experiments were performed at an approved institutional Good Manufacturing Practice (GMP) facility (Cell Factory, Fondazione IRCCS Policlinico San Matteo) in the allogeneic setting. Three batches of donor-derived memory-like NK cells were produced from 5 × 10^8^ PBMCs derived from haplo-HSCT donor leukaphereses with experimental Protocol B. At the end of the culture, memory-like NK cells were cryopreserved in different vials. For the GMP validation, cell recovery, vitality, phenotype and potency were evaluated before and after cryopreservation. In addition, batches of memory-like NK cells were subjected to quality control (QC) in compliance with GMP requirements. Microbiological checks of cellular products were carried out under aseptic conditions according to European Pharmacopoeia (EP) protocols as previously described in detail [31]. The identity of each batch genotype was confirmed with a previously described PCR-based assay analyzing short tandem repeat (STR) loci [32].

### 2.10. Statistical Analysis

The *t*-test for paired data was used to compare the different in vitro conditions. Results were reported as the means and standard deviations (SD). A *p*-value < 0.05 was considered significant.

## 3. Results

### 3.1. Establishment of Optimal Culture Conditions for the Induction of Memory-Like NK Cells with Strong Anti-Tumor Activity

In the first part of the study, we compared different approaches to activate and expand NK cells with memory-like features endowed with high levels of anti-tumor activity and with appreciable cell recovery at the end of the cultures. We used purified NK cells obtained from buffy coats of five healthy donors or from leukaphereses of two haplo-HSCT donors, and their cytotoxic activity was measured against the THP-1 cell line or the leukemia blasts (LB) derived from one ALL patient and one AML patient at the end of the different culture conditions.

NK cells were activated for 16 h with a cocktail containing IL-2, IL-15 and IL-18 as previously described (18) and then maintained for 6–7 days with a different combination of cytokines (Figure 1A, right panel). As reported in Figure 1B, compared to the initial culture (day 0), 16 h of cytokine activation (day 1) caused a marked reduction of cell recovery in all tested donors (mean, 60 ± 8%). In the subsequent maintenance phase, we compared the four different culture conditions listed in Figure 1A (namely, Protocols A, B, C and D). The data summarized in Figure 1C,D show that the concentration of IL-2 and its combination with IL-15 were critical to both cell recovery and cytotoxic activity achieved at the end of the culture. Indeed, the use of a low IL-2 concentration (10 U/mL, protocol A) with IL-15 or of IL-15 alone (protocol D) resulted in a low recovery of NK cells displaying high anti-leukemia lytic levels assessed against the THP-1 cell line, whereas a higher concentration of IL-2 combined with IL-15 was associated with a progressively enhanced cellular recovery but a decreased cytolytic activity of NK cells. In these culture conditions, the extension of the maintenance period to 14 or 21 days decreased the NK cell recovery, while the levels of cytotoxic activity against leukemia remained quite similar to that observed at day 7 (Figure 1C,D). Comparable cytolytic activity against THP-1 cells was obtained using NK cells derived from either buffy coats of healthy donors (Figure 1D) or using leukaphereses of haplo-HSCT donors against the respective recipient’s LBs (Figure 1E). Notably, activated memory-like NK cells comparably killed AML or ALL blasts. These data suggest that Protocol B provided optimal culture conditions yielding the highest number of NK cells endowed with the most efficient killing capacity, and therefore it was utilized thereafter.

We next measured cell recovery and anti-leukemia activity of NK cells after 6 days with the B culture protocol with or without a further 6-h reactivation and 16-h maintenance (Figure 2A). The results were compared with those obtained with NK cells activated with 100 U/mL of IL-2 (control NK). We documented that the reactivation of NK cells caused a loss of cell recovery (Figure 2B) without any increase in cytotoxic activity against the THP-1 cell line compared with the NK cells that were not reactivated (Figure 2C). The level of control NK cytotoxic activity cell against THP-1 cells was always significantly lower than that of in vitro activated and expanded NK cells (Figure 2C).

### 3.2. Activated NK Cells Exhibit a Memory-Like Phenotype

**Figure 3:**
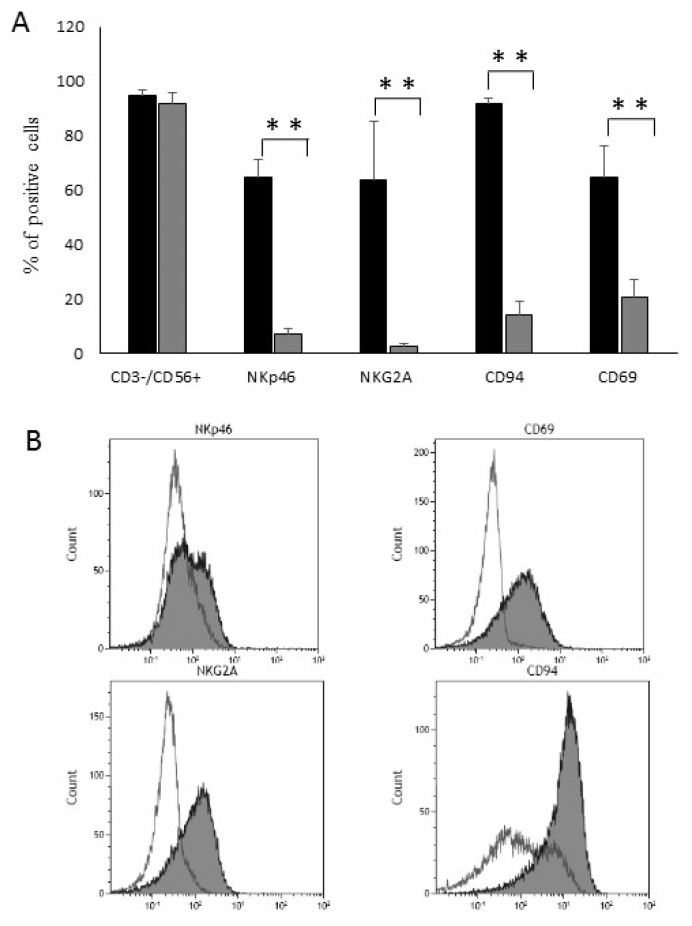
Phenotypic evaluation. (**A**) Analysis of surface antigen expression on control NK cells and memory-like NK cells obtained with protocol B. Results are expressed as the means and standard deviation (SDs) of the receptor surface expression percentages, ** *p* < 0.01. (**B**) Representative results showing the up-modulation of surface antigen expression from day 0 (white profiles) to day 7 (grey profiles).

NK cells obtained using Protocol B were characterized by a marked upregulation of CD69, NKG2A and CD56 (Figure 3A,B), clearly indicating that they expressed a memory-like phenotype (as described in 18). Together, these results confirmed that we had defined optimal culture conditions for the expansion of strong lytic anti-leukemia NK cells with a memory-like phenotype.

### 3.3. Persistence of Memory-Like NK Cells in NSG Immunodeficient Mice

**Figure 4:**
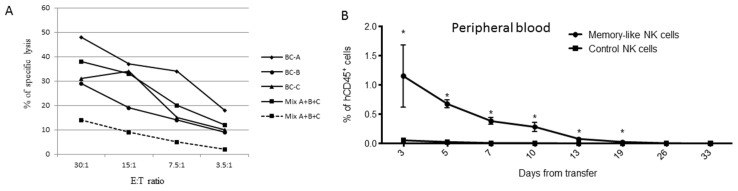
Evaluation of NK cell persistence in mice. NK cells were isolated from buffy coats of three donors, activated using Protocol B (memory-like NK cells) or, alternatively, with rhIL-2 Overnight (control NK). At the end of the culture, the cells were cryopreserved and after thawing their potency was confirmed against THP-1 cell lines before transferring them into mice. (**A**) Cytotoxic activity of memory-like NK cells derived from buffy coats of three healthy donors (BC-A, BC-B and BC-C) and from a mix of the three buffy coats (Mix A + B + C). The dashed line represents the cytotoxic activity of NK cells from a mixture of buffy coats activated ON with rhIL-2 (control NK). (**B**) Kinetics of recovery of memory-like and control NK cells after transfer into mice. The recovery of memory-like NK cells was higher than that of control NK starting from day +3 to day +19 after in vivo transfer (* *p* < 0.01).

To evaluate whether the memory-like NK cells generated with Protocol B were able to persist longer upon in vivo transfer compared with the NK cells activated ON with 100 U/mL of IL-2, we transferred 7 × 10^6^ NK cells/mouse into immunodeficient NSG mice and followed the presence of circulating human CD45^+^CD56^+^CD3^−^ cells by bleeding the mice every two days. To reach the number of cells required for transfer into four NSG mice per group, the NK cells were purified from three different buffy coats and either expanded separately or pre-mixed before in vitro expansion. Prior to transfer into mice, their cytotoxic activity was tested against THP-1 cells. The data reported in Figure 4A confirmed the higher level of killing by the memory-like NK cell populations either derived from single buffy coats or from the mixtures compared to the control NK cells. The in vivo persistence of the memory-like NK cells was significantly longer compared to the control cells (Figure 4B and Figure S1). Notably, we identified human CD45^+^ cells expressing CD56 and CD94 markers up to 19 days after the transfer, whereas the control NK cells persisted for less than one week. These data suggested that memory-like NK cell populations were endowed with longer in vivo persistence than the control ones, strongly supporting their suitability for adoptive immunotherapy.

### 3.4. Haploidentical Donor-Derived Memory-Like NK Cells Efficiently Kill Patients’ Leukemic Blasts

**Figure 5:**
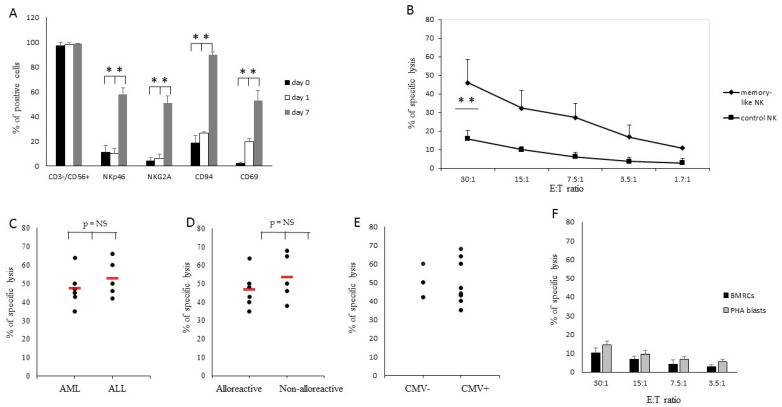
Induction of memory-like NK cells in haploidentical HSCT donors. (**A**) Phenotypic evaluation of surface antigens of donor-derived memory-like NK cells at day 0 (after isolation from donor leukapheresis), at day 1 (after 16-h activation) and at day 7 (at the end of the culture). (**B**) Cytotoxic activity of memory-like (♦) or control (■) NK cells was assessed using patients’ Leukemia Blasts (LBs) as target cells. Cytotoxicity was evaluated in a 4-h chromium release assay at different E:T ratios. Memory-like NK cells derived from haplo-HSCT donors were assessed respectively against patients’ LBs. The means and SDs are reported, ** *p* < 0.01. (**C**) Results of specific cytotoxic activity of alloreactive and non-alloreactive donor-derived memory-like NK cells against patients’ LB (E:T ratio of 30:1). The red line represents the mean cytotoxic activity. (**D**) Results of the specific cytotoxic activity of donor-derived memory-like NK cells against patient AML and ALL LBs (E:T ratio of 30:1). The red line represents the mean cytotoxic activity. (**E**) Levels of specific cytotoxic activity displayed by memory-like NK cells with respect to the CMV status of the haplo-HSCT donor. (**F**) The means and SDs of cytotoxic activity of memory-like NK cells against patients’ non-malignant cells, namely, bone marrow remission cells (black columns) or PHA blasts (grey columns).

Having demonstrated that the NK cells obtained with Protocol B have a memory-like phenotype and the ability to persist longer in mice compared to the control NK cells, we applied protocol B to the generation of donor-derived memory-like NK cells in a haplo-HSCT setting. Haplo-HSCT recipients were affected by AML (4 pts) or by ALL (6 pts) and underwent T cell-depleted transplantation from haploidentical family donors (Table 1). After 7 days of ex vivo culture with Protocol B, donor-derived NK cells acquired a memory-like phenotype with the upregulation of CD94, NKG2A, NKp46 and CD69 (Figure 5A, grey column; *p* < 0.01) compared with the same cells stained directly ex vivo (black column) or after the 16 h of activation (white column) (*p* < 0.05 for all surface antigens) (Figure 5A). The killing ability of donor-derived memory-like and control NK cells was tested in a 4-h cytotoxicity assay against the LBs, PHA blasts and bone marrow remission cells (BMRCs) derived from each patient. The results showed that the control NK cells were less lytic against patients’ LBs at all the E:T ratios evaluated (mean: 16 ± 4% at an E:T ratio of 30:1), whereas memory-like NK cells were endowed with a high level of anti-leukemia cytotoxic activity (mean: 46 ± 12% at an E:T ratio of 30:1; *p* < 0.05) (Figure 5B). As already observed in two donor/recipient pairs (Figure 1E), memory-like NK cells isolated from haplo-HSCT donors showed comparable cytolytic activity against myeloid or lymphoblastic LBs (Figure 5C), whereas their killing of patient-derived PHA blasts or BMRCs was always negligible (less than 15%) at all E:T ratios. Next, we investigated whether the presence of an alloreactive NK cell population positively influenced the ability of donor-derived memory-like NK cells to kill LBs. To this aim, we investigated NK alloreactivity according to the KIR/KIR ligand mismatch in the graft-versus-host direction model and identified six out of 11 donors characterized by a *KIR* gene coding for an inhibitory receptor recognizing a KIR ligand present in the donor and absent in the patient. In particular, one donor had a C1 NK alloreactive population, four—a C2 and one—a Bw4 (Table 1 and Figure S2). We documented that memory-like NK cells derived from both alloreactive and non-alloreactive donors had high levels of cytotoxicity against patients’ LBs (Figure 5D). In addition, the CMV status of the haplo-HSCT donor did not affect the levels of cytotoxicity displayed by memory-like NK cells (Figure 5E). Low levels of cytotoxic activity against patients’ non-malignant cells were documented at all E:T ratio (Figure 5F). In the haploidentical setting, the percentage of residual T cells in memory-like NK cell preparations is of critical importance since the former cells could induce GvHD once infused into the recipients.

The data reported in Table 3 show that the percentage and absolute number of CD3^+^ T cells was significantly reduced after 7-day culture compared to that observed at day 1. After NK activation, there was a marked reduction in the total number of cells and a modest decrease in the absolute number of CD3+ T cells, but high levels of cytotoxicity against patients’ LBs. Again, after 7-day culture, we observed a relative reduction in the percentage and absolute number of residual CD3^+^ T cells. Analysis of surface antigens demonstrated an upregulation of the natural cytotoxic receptors (NCRs) and of a number of adhesion/homing surface molecules such as CD38, CD18, CD49L, CD62L and CXCR4 in the memory-like NK cells compared to the control NK cells (Table 4).

### 3.5. Memory-Like NK Cells Derived from Cancer Patients Efficiently Kill Autologous Tumor Cells

**Figure 6:**
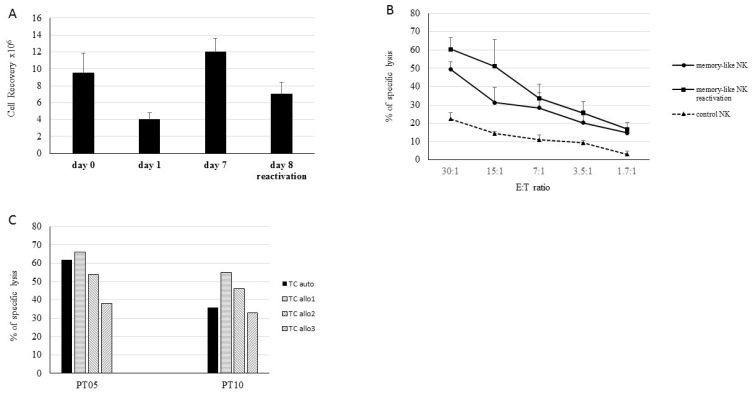
Induction of memory-like NK cells in cancer patients. NK cells derived from PBMCs of five cancer patients were activated with IL-12, IL-15 and IL-18 followed by 6-day culture in the presence of IL-2 (40 U/mL) and IL-15 (1 ng/mL). After 6 days in the maintenance phase, the cells were recovered or alternatively reactivated. (**A**) The means and SDs of cell recovery of memory-like NK cells derived from PBMCs of cancer patients at various timepoints of cultures. (**B**) The means and SDs of cytotoxic activity of NK cells obtained at day 7 (♦), at day 8 (■) and of control NK cells (activated ON with rhIL-2) (▲) are reported. Memory-like and control NK cells were tested against autologous TCs at various E:T ratios. (**C**) Representative cytotoxic activity experiments of memory-like NK cells derived from two cancer patients tested against autologous (black columns) or allogeneic (striped columns) TCs derived from the same type of cancer, sarcoma (PT-05) or RCC (PT-10), respectively.

The induction of autologous memory-like NK cells was then evaluated in patients with advanced solid tumors (Table 2). NK cells purified from patients’ PBMCs were either stimulated ON with 100 U/mL of rhIL-2 (control NK) or activated and maintained for 6 days using Protocol B (memory-like NK cells). The recovery of purified NK cells from the peripheral blood of patients varied, but the rate of their expansion after the activation and maintenance phases was in line with that observed in the experiments performed with NK cells from healthy donors (Figure 6A). Indeed, we observed a decrease in their number after activation (day 1) followed by substantial cell recovery during the maintenance phase. Memory-like NK cells derived from patients displayed high levels of specific cytotoxic activity against autologous TCs, significantly higher than that displayed by control NK cells (*p* < 0.05) (Figure 6B). In patient-derived memory-like NK cells, reactivation at day 7 increased the levels of lysis (although not significantly, *p* > 0.05) associated with a decrease in the number of viable cells. The level of cytotoxicity against autologous TCs was comparable to that obtained against the allogeneic TCs derived from the same type of cancer, sarcoma or Renal Cell Carcinoma (RCC), respectively (Figure 6C). Phenotypic analysis confirmed that the memory-like NK cells from cancer patients, like donor-derived memory-like NK cells, also displayed overexpression of NCRs and of adhesion/homing surface molecules compared to the control NK cells (Table 4).

### 3.6. Evaluation of IFNγ-Secreting NK Cells

**Figure 7:**
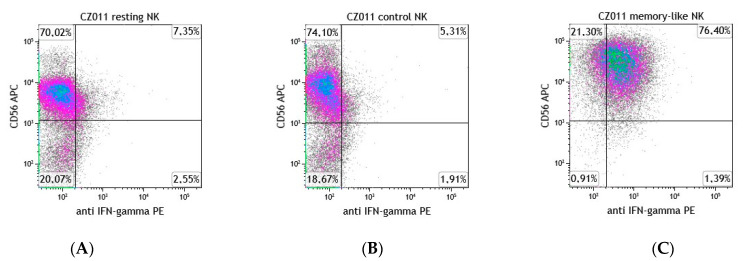
Memory-like NK cells exhibit a high percentage of IFNγ-secreting cells. Flow cytometry plots of IFNγ-secreting cells from control (**A**) resting (**B**) and memory-like NK cells (**C**) derived from one representative haplo-HSCT donor.

The memory-like NK cells derived from haplo-HSCT donors or from cancer patients were evaluated for their capacity to secrete IFNγ in comparison with the control NK cells. We documented a high percentage of the memory-like NK cells capable of secreting IFNγ compared to that observed in the control NK cells (Table 5 and Figure 7).

### 3.7. GMP Expansion and Validation

Three batches of donor-derived memory-like NK cells were produced at the GMP facility of the Institution from PBMCs derived from haplo-HSCT donor leukaphereses according to GMP guidelines. In all experiments, the initial number of PBMCs was 5 × 10^8^. After 16-h activation and 6-day expansion, memory-like NK cells underwent microbiological and biological QC and were cryopreserved. QC testing was repeated on memory-like NK cells after thawing. Recovery, vitality and potency of memory-like NK cells were in line with that reported above in haplo-HSCT donors. In particular, the levels of cytotoxicity against recipient patients’ LBs before and after cryopreservation were comparable (Table 6). Significant differences in pre- or post-thaw cells were not observed in the expression of all evaluated surface antigens (data not shown). Both microbiological and biological controls confirmed that memory-like NK cells were suitable for transfer to patients. Based on these data, we can extrapolate that, starting with 10 × 10^9^ leukapheresis donor PBMCs, it should be possible to cryopreserve at least 1 × 10^9^ memory-like NK cells suitable for adoptive immunotherapy approaches.

## 4. Discussion

The success of anti-tumor adoptive cell therapy depends on many factors, such as the rapid generation of highly effective anti-tumor lymphocytes and their expansion to clinically relevant quantities. Therefore, one of the major prerequisites for anti-tumor cell therapy is the possibility of generating and expanding ex vivo patient- or donor-derived immune cells with high levels of anti-tumor activity. Multiple methods are being explored on how to enhance the potency of NK cells against hematological or solid malignancies and, among these, one of the most promising is the ex vivo induction of memory-like NK cells. With the aim of addressing these aspects, our study initially focused on defining the best experimental conditions to expand adequate numbers of memory-like NK cells while preserving their anti-tumor activity by adapting the methodology previously described by Romee et al. [21]. The authors documented that memory-like NK cells could be cultured with low-dose IL-15 to support survival for 7, 14 or 21 days. However, in our hands, using only IL-15 in the maintenance phase, the recovery of cells at the end of the cultures was too low, so we decided to evaluate the use of different doses of IL-2 in association with IL-15 to sustain cultures. In preliminary experiments, the potency of NK cells derived from buffy coats of healthy donors was tested against the THP-1 AML cell line. After 16-h activation with IL-12, IL-15 and IL-18, a marked reduction in the number of viable NK cells was documented, while the dose of IL-2 used in combination with 1 ng/mL of IL-15 during the maintenance/expansion phase (from day 1 to day 7) was critical to the recovery and anti-tumor activity of NK cells. IL-2 and IL-15 are known to stimulate proliferation, survival and functional activities of NK cells [33,34]; for this reason, their use in the maintenance phase could be useful for sustaining the expansion of cytotoxic NK cells. We showed that low doses of rhIL-2 or rhIL-15 alone were able to induce memory-like NK cells endowed with potent anti-tumor activity; however, the recovery of viable cells obtained at the end of the cultures was very low, thus, unsuitable for adoptive cell therapy approaches. On the contrary, high-dose rhIL-2 induced a marked increase in the number of NK cells, albeit with a reduction in their killing potency, probably because of the expansion of a subset of NK cells with modest killing activity against malignant cells.

Using NK cells purified from buffy coats from healthy donors, similar results were confirmed in the setting of allogeneic HSCT when NK cells purified from haploidentical donors were tested against patients’ leukemic myeloid or lymphoid blasts. It is important to underscore that, in all the conditions evaluated, the efficacy of the memory-like NK cells was significantly higher than that observed in the control NK cells activated with IL-2. Most adoptive NK-based clinical trials aim to control cancer using allogeneic or autologous NK cells after isolation and, in some cases, a short in vitro activation with cytokines [35]. This strategy results in a short-term priming that might increase NK cells’ functional capacity, but the effect is rapidly lost after removal from the in vitro cytokine milieu and transfer into patients. As previously described in a murine model [36], we demonstrated that cytokine re-stimulation or extension of the culture time up to three weeks is not necessary for maintaining memory-like NK cells and that the initial 16 h of activation could induce a stable memory-like state. Evaluation of their transfer in the murine model clearly demonstrated their capacity to persist in vivo for at least three weeks, much longer than the control NK cells which are detectable for only few days in the peripheral blood of injected mice do, in agreement with the data showing that the half-life of NK cells is about seven days [37].

After having established an optimal approach for expanding memory-like NK cells while preserving their anti-tumor activity, we demonstrated that memory-like NK cells could be induced from both peripheral blood of haploidentical donors in the setting of HSCT and from cancer patients. We showed that donor-derived memory-like NK cells with the ability of efficiently killing patients’ LBs showed negligible activity against patients’ non-malignant cells and could be successfully induced from NK cells isolated from donor leukaphereses. Notably, the potency of donor-derived memory-like NK cells did not depend on donor NK alloreactivity. Indeed, similar levels of anti-leukemia activity were obtained using memory-like NK cells derived from alloreactive and non-alloreactive NK donors.

In addition, no significant differences were observed in the levels of cytotoxic activity when target LB cells were derived from AML or ALL patients. Nevertheless, retrospective studies have clearly documented that the presence of donor NK alloreactivity correlates with a better clinical outcome in both adult AML patients and pediatric ALL patients [6,7,8,38,39,40]. Thus, when more alternative donors are present, the donor NK alloreactivity represents a positive selection criterion. NK alloreactivity occurs in only around 50% of donor/recipient pairs [40], so even if additional criteria for the selection of the best donors have been added in the last few years, about half of haplo-HSCT recipients lack the alloreactivity effect mediated by NK cells. It is well-known that the prognosis for children transplanted with a haploidentical family donor in an advanced disease phase or in the presence of a measurable minimal residual disease (MRD) or of unfavorable cytogenetic abnormalities is still poor, especially in patients who cannot benefit from the expansion of alloreactive donor NK cells with anti-leukemia activity immediately after transplantation. For high-risk patients, the availability of donor-derived memory-like NK cells able to persist in vivo and to efficiently kill both myeloid and lymphoblastic leukemia cells but unable to induce GvHD may overcome the lack of donor alloreactivity (or enhance this effect) leading to the control of leukemia relapse in the early post-transplant period. In humans, elevated frequencies of memory-like NK cells that include a variety of NK cell subsets expressing different receptors have been documented in association with Human Cytomegalovirus (HCMV) infection [18,41]. For this reason, it might be interesting to evaluate the possible advantage in deriving memory-like NK cells from CMV-positive individuals. Even though the majority of haplo-HSCT donors evaluated were CMV-positive, our data suggest that the CMV status of the donor did not affect the in vitro induction of memory-like NK cells and their levels of anti-leukemia cytotoxic activity. To address this point, a phase I clinical trial is ongoing to evaluate the effect of infusing memory-like NK cells from a donor previously exposed to CMV in association with IL-2 and chemotherapy [42].

The potential efficacy of haploidentical memory-like NK cells to mediate an anti-leukemia response has already been documented in a first-in-human phase I trial in patients with active relapsed/refractory AML [23]. Escalating doses of haploidentical memory-like NK cells were transferred in preconditioned (fludarabine/cyclophosphamide) AML patients after 16-h activation with IL-12, IL-15 and IL-18 [21]. This promising study observed clinical responses, including complete remission in older AML patients after multiple previous treatments. At present, other ongoing clinical trials are evaluating the efficacy of infusing cytokine-induced memory-like NK cells in the setting of allo-HSCT for AML in association with Donor Lymphocytes Infusion (DLI) or using ALT-803 as an alternative for IL-2 [42,43]. In the present study, we evaluated the use of donor-derived memory-like NK cells in the setting of haplo-HSCT and confirm that these cells were active in vitro against both AML and ALL blasts from pediatric patients. These data provide evidence that the therapeutic efficacy of memory-like NK cells is not limited to AML but is extendable to B-ALL not only in adults, but also in pediatric patients. We documented that prolonging the culture for a further six days (maintenance phase) led to an increased number of cells at the end of the culture and significantly reduced the number of residual CD3 cells in the culture. This is important because, unlike T cells, NK cells are, in general, unable to induce GvHD, thereby allowing their transfer from the donor to the recipient in an allogenic setting [44]. Only a few studies have reported the development of acute GvHD after transfer of allogeneic NK cells, but the relationship between NK cells and GvHD is still unclear. Few data are available at present regarding the impact of infusing memory-like NK cells on the development of GvHD in humans, while experiments in murine models suggest that cytokine-induced memory-like NK cells appear to protect from severe GvHD in most cases [42]. It should also be emphasized that after infusion of NK cell populations, there were other factors possibly contributing to this adverse event, in particular, the presence of residual T cell contamination. [45,46,47]. Here, we documented that the absolute number of residual T cells in the final product significantly declined when the activation phase was combined with the maintenance phase. These data, together with the evidence that memory-like NK cells display low levels of cytotoxicity against patients’ nonmalignant cells (i.e., BM remission cells or PHA blasts), suggest that these cells are at very low risk of inducing GvHD after transfer into patients.

Concerning therapeutic use of autologous NK cells in patients with solid malignancies, a number of studies have demonstrated that the infusion of NK cells is safe and well-tolerated, although there is little evidence supporting the real effectiveness of NK cells in eradicating autologous tumors [48]. Many studies indicate that NK cells from cancer patients might have altered phenotype and function with a poorer capacity to recognize and control tumor cells [12,49], while the infusion of haploidentical NK cells has a limited role in the reduction of tumor burden in patients with solid tumors [48]. Consequently, it is encouraging that the autologous memory-like NK cells obtained with the culture conditions described in this study possess not only the capacity to persist longer in vivo than the NK cells activated ON with rhIL-2, but also display very high levels of anti-tumor activity in vitro. These data also suggest that in vitro induction of autologous memory-like NK cells endowed with high anti-tumor activity was not impaired by previous immunosuppressive therapies to which patients had been subjected.

The number of autologous NK cells obtained at the end of the culture is compatible with the adoptive immunotherapy of cancer patients which typically requires a high number of cells [47]. The infusion of autologous NK cells endowed with potent anti-tumor activity and able to persist for long periods in vivo may hence overcome the reduced functionality of autologous cells. In addition, transfer of autologous NK cells does not require any immunosuppressive treatment, thus preserving NK cell functions. As already described in murine and human memory-like NK cells [18,21,22], we documented that the majority of memory-like NK cells derived from both haplo-HSCT donors and cancer patients were able to secrete IFNγ compared with the low percentage present in control NK cells. This information is important in that it is known that, although cytotoxic activity is the major function of NK cells, IFNγ production also plays an important role in determining the effector functions as IFNγ exerts antiproliferative and proapoptotic effects on tumor cells. A previous study demonstrated that the expression of activating receptors could be enhanced in NK cells by IL-2 treatment. In our study, we documented that in the memory-like NK cells from healthy donors and cancer patients, there is an increase in the expression of the NCRs, NKp30, NKp44 and NKp46 superior to that induced by treatment with IL-2 alone in the control NK cells. NCRs have been proposed to bind to many cellular ligands that are implicated in NK cell surveillance of tumor cells. Many of these interactions have been shown to evoke the cytotoxic and cytokine-secreting functions of NK cells. This is in agreement with the data of this study where we documented high levels of cytotoxic activity and IFNγ secretion displayed by memory-like NK cells [50].

Memory-like NK cells derived from both haplo-HSCT donors and cancer patients also display some adhesion/homing surface antigens such as CD38, CD18, CD49D, CD62L and CXCR4, which could favor their proliferative and migration capacity and have a role in mediating cytotoxicity against tumor cells [51,52,53,54]. Our study suggests that NK memory-like cells can be readily produced and that after cryopreservation and thawing they maintain their features and potency. Considering that only one week of culture is required to generate memory-like NK cells, this approach could be transferred to future clinical trials of adoptive NK cell therapy.

## 5. Conclusions

Here, we demonstrated the feasibility of obtaining the autologous or donor-derived memory-like NK cells displaying high levels of anti-tumor activity and low levels of reactivity against non-malignant cells that were able to persist much longer in mice than the control NK cells. This approach could be readily transferred to future clinical trials of adoptive NK cell therapy in high-risk pediatric patients affected by acute leukemia in the early period after haplo-HSCT or in refractory cancer patients in progression after having failed conventional therapies.

## Figures and Tables

**Table 1:** Characteristics of donor/recipient pairs in the haploidentical setting.

				NK Alloreactivity ^£^	
UPN	Disease ^¶^	Donor	Donor CMV ^§^ Status	Yes/No (Type)	Relevant KIR
LM004	AML	Mother	positive	Yes (C1)	KIR2DL2/L3
LR001	AML	Sister	negative	Yes (C2)	KIR2DL1
GM005	AML	Mother	negative	Yes (C2)	KIR2DL1
UT006	ALL	Mother	positive	Yes (C2)	KIR2DL1
CR008	ALL	Mother	positive	Yes (C2)	KIR2DL1
CM002	ALL	Mother	positive	Yes (Bw4)	KIR3DL1
SQ003	ALL	Mother	positive	No	n.a. ^&^
SC009	ALL	Mother	positive	No	n.a.
AV010	AML	Mother	positive	No	n.a.
CZ011	ALL	Mother	positive	No	n.a.
BF007	AML	Mother	negative	No	n.a.

^£^ Presence of Natural Killer (NK)alloreactivity was assessed using the Killer-cells Immunoglobulin-like Receptors (KIR) KIR/KIR ligand mismatch in the graft-versus-host direction model. ^&^ Not applicable. ^¶^ Acute Myeloid Leukemia (AML), Acute Lymphoblastic Leukemia (ALL); ^§^ Cytomegalovirus (CMV).

**Table 2:** Clinical characteristics of patients with solid tumors included in the study.

UPN	Disease	Source of Tumor Cells	Previous Therapies
PT01	Renal cell carcinoma Lung and lymph node metastases	Lung metastases	Nephrectomy, 2005 Vinblastine–IFN, 2008 Sunitinib, 2008–2010 Everolimus, 2010–2011 Sorafenib, 2011 IFN, 2012
PT03	Renal cell carcinoma Lung metastases	Lung metastases	Nephrectomy, 2010 Pazopanib, 2012 Everolimus, 2012–2013 Sorafenib 2013
PT05	Retroperitoneal sarcoma Liver metastases	Liver metastases	IFO–adriamycin, 2011 Docetaxel–gemcitabine, 2012
PT06	Seropapillary ovarian cancer BRCA-mutated Stage IIIC	Peritoneum	Debulking surgery, CarboTaxol, 2012 Liposomal doxorubicin, 2014 Topotecan, 2014
PT010	Renal cell carcinoma Lung and bone metastases	Lung metastases	Nephrectomy, 1995 IL2, 2007 Sorafenib, 2007–2009 Sunitinib, 2009–2010 Everolimus, 2010–2011

**Table 3:** Residual CD3-positive cells in donor-derived memory-like NK cells.

	Day 0 Mean (Range)	Day 1 Mean (Range)	Day 7 Mean (Range)
Cell recovery * (×10^6^)	8.2 (7.2–9.5)	2.1 (1.8–2.0)	4 (3.6 (3.6–4.8)
CD3-neg/CD56+, %	94.3 (94–95)	92 (88–95)	99 (98.5–99.2)
CD3+, %	1 (0.5–1.5)	2.7 (1.7–3.7)	0.2 (0.1–0.3)
CD3+ absolute number (×10^4^)	8.5 (3.6–14)	5 (2.4–7.4)	0.7 (0.36–1)
% of specific lysis ^§^	5 (2–8)	57 (25–74)	43 (28–56)

* NK cells were purified starting from 1 × 10^8^ donor leukapheresis. ^§^ Percentage of specific lysis of NK cells against patients’ LBs in a 4-h cytotoxicity assay at an E:T ratio of 30:1.

**Table 4:** Surface phenotype of memory-like NK cells and control NK cells.

Surface Receptors	Memory-Like NK Cells		Control NK	
	Haplo- Donors ^§^	Cancer Patients	Haplo- Donors	Cancer Patients
**NKp30**	39 (35–44)	42 (36–45)	5 (3–6)	7 (5–8)
**NKp44**	47 (41–49)	45 (40–47)	3 (1–4)	5 (4–9)
**NKp46**	62 (60–64)	68 (65–73)	7 (6–9)	7 (5–10)
**NKG2A**	61 (57–64)	70 (68–72)	2 (1–4)	5 (3–8)
**CD18**	98 (98–99)	97 (95–98)	98 (97–99)	98 (97–99)
**CD38**	97 (96–98)	97 (96–99)	86 (80–89)	84 (80–86)
**CD49d**	98 (98–99)	97 (94–99)	90 (87–94)	88 (83–91)
**CD62L**	28 (25–32)	35 (32–39)	15 (10–18)	19 (17–24)
**CD69**	64 (60–70)	60 (35–44)	20 (18–23)	12 (10–13)
**CD94**	92 (90–95)	90 (35–44)	22 (17–25)	20 (17–28)

^§^ For each surface antigen, the median and the range are reported. The surface antigen percentage refers to CD3-neg/CD56+ cells.

**Table 5:** IFNγ-secreting cells.

	Haplo-Donor ^§^	Cancer Patients
Memory-like NK cells	76 (75–88)	73 (70–86)
Control NK cells	7 (6–12)	9 (7–14)

^§^ The median percentages and ranges are reported. Results refer to memory-like NK cells and control NK cells derived from three different haplo-HSCT donors and cancer patients.

**Table 6:** Scale-up experiments for GMP validation.

UPN	PBMC *	% CD3–/CD56+ *	CD3–/CD56+ *	16-h Activation*	7-Day Culture * (Final Product)	% Specific Lysis before Cryopreservation ^¶^	% Specific Lysis after Thawing
SC009	5.0 × 10^8^	10%	3.0 × 10^7^	1.8 × 10^7^	4.7 × 10^7^	55	58
CM002	5.0 × 10^8^	12%	6.2 × 10^7^	5.1 × 10^7^	8.3 × 10^7^	47	40
GM005	5.0 × 10^8^	15%	6.6 × 10^7^	4.4 × 10^7^	8.5 × 10^7^	43	46

* cell recovery, ^¶^ percentage of specific lysis against patients’ LB at an E:T ratio of 30:1. After 7-day culture, the vitality of memory-like NK cells ranged between 91–95%.

## Data Availability

Data sharing not applicable.

## Supplementary Materials

The following are available online at https://www.mdpi.com/article/10.3390/cancers13071577/s1, Figure S1 Gating strategy; Figure S2: Analysis of NK alloreactive donors.
