# Supplementary material for: Cytokine-Induced Memory-Like NK Cells with High Reactivity against Acute Leukemia Blasts and Solid Tumor Cells Suitable for Adoptive Immunotherapy Approaches
Source: Cancers (Basel). 2021 Mar 30;13(7):1577. doi: 10.3390/cancers13071577 (PMC8036252; doi:10.3390/cancers13071577)
Supplement: Supplementary file 1 [file cancers-13-01577-s001.pdf]

Supplemental Figure 1: Gating strategy.

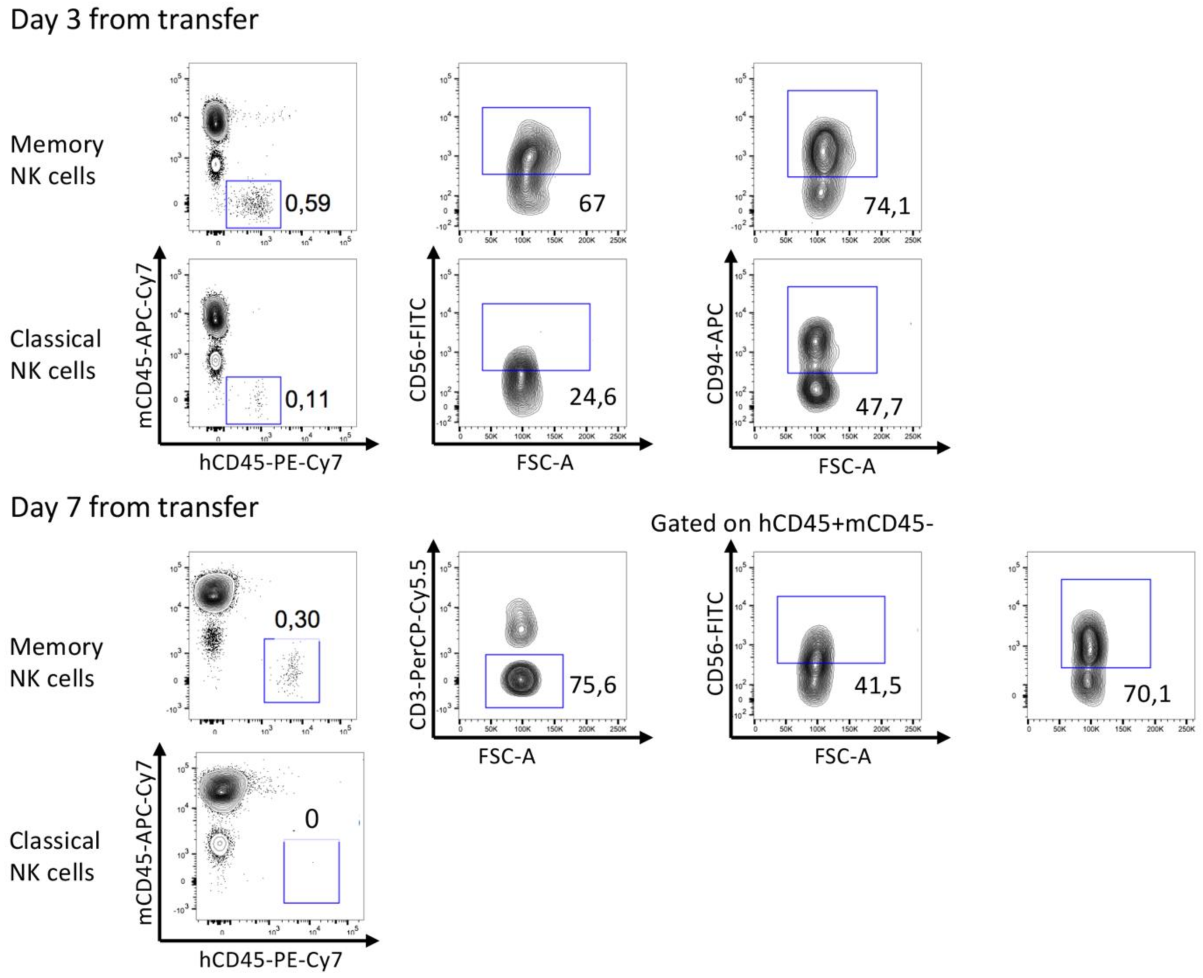

A

	patient KIR-L				donor KIR-L			
UPN	C1	C2	B Bw4	A Bw4	C1	C2	B Bw4	A Bw4
LM004		*05:01, *15:02	*51:01		*01:02	*05:01		
LR001	*08:02,*08:02			*24:02	*08:02	*04:01		*24:02
GM005	*07:01,*07:02		*49:01	*32:01	*07:01	*02:02	*49:01,*51:01	*32:01
UT006	*01:02,*12:03			*24:02	*01:02	*04:01	*51	*24:02
CR008	*01:02,*07:02		*27:05		*07:02	*04:01		
CM002	*03:04,*07:02				*07:02,*12:02		*52	
SQ003	*08:02	*06:02	*53:01	*32:01		*05:01, *06:02	*53:01	*32:01
SC009	*12:03	*06:02	*38:01,*57:01		*07:02	06:02	*57:01	
AV010	*03:03,*14:02		*51:01	*32:01	*03:03,*07:02			*32:01,*32
CZ011	*12:03	*02:02		*24:02		*02:02,*05:01		
BF007	*03,*08			*23	POS[£]	NEG[£]		*23,*32

B

	KIR repertoire analysis																			
UPN	3DL3	2DS2	2DL2	2DL3	2DL5B	2DS3	2DP1	2DL1	3DP1	RS	2DL4	3DL1	3DS1	2DL5A	2DS3/S5	2DS1	2DS4	3DL2	NK alloreactivity	relevant KIR
LM004						S3						T			S5		D		YES (C1)	KIR2DL2/L3
LR001												C					F		YES (C2)	KIR2DL1
GM005												C			S5		D		YES (C2)	KIR2DL1
UT006												C					F,D		YES (C2)	KIR2DL1
CR008												C			S3		D		YES (C2)	KIR2DL1
CM002												C			S5		F		YES (Bw4)	KIR3DL1

Supplemental Figure 2: Analysis of NK alloreactive donors. **(A)** Analysis of KIR ligand in 11 donor/recipient pairs. HLA class I alleles were converted in KIR ligand using the free KIR-ligand calculator program (https://www.ebi.ac.uk/ipd/kir/ligand.html). Data were integrated including HLA-A*23, -A*24, and -A*32 in the allotypes recognized by KIR3DL1 receptor (1). When present, the HLA allele coding for the KIR ligand present in the donor and absent in the recipients (i.e. relevant for NK alloreactivity) were boxed in gray. [£] For donor BF007, since HLA-C typing was not available, positivity for HLA-C C1 and negativity for HLA-C C2 alleles were assigned using SSP-PCR approach. **(B)** *KIR* repertoires of NK alloreactive donors were analysed for either the presence (grey boxes) or the absence (white boxes) of the indicated *KIR* genes. S3 and S5 in the boxes indicate the presence of *KIR2DS3* and *KIR2DS5*, respectively. In *KIR2DS4* boxes, F indicates the presence of allele(s) coding for membrane bound receptor, while D indicates allele(s) coding for putative soluble receptor(s). *KIR3DL1* alleles coding for functional receptors or for polypeptides retained into the cell are reported with T and C, respectively, according to the nucleotide present at position 320 (2). The gene order was established on *KIR* haplotype published sequences. Dark grey boxes indicate the *KIR* genes relevant for NK alloreactivity. Recombination hotspot site, located between centromeric and telomeric regions, is indicated with RS.

References

1 Human leukocyte antigens A23, A24, and A32 but not A25 are ligands for KIR3DL1. Stern M, Ruggeri L, Capanni M, Mancusi A, Velardi A. Blood. 2008 Aug 1;112(3):708-10. doi: 10.1182/blood-2008-02-137521. Epub 2008 May 23. PMID: 18502829

2 Hematopoietic stem cell transplantation: Improving alloreactive Bw4 donor selection by genotyping codon 86 of KIR3DL1/S1. Alicata C, Pende D, Meazza R, Canevali P, Loiacono F, Bertaina A, Locatelli F, Nemat-Gorgani N, Guethlein LA, Parham P, Moretta L, Moretta A, Bottino C, Norman PJ, Falco M. Eur J Immunol. 2016 Jun;46(6):1511-7.
